# A Stable RNA Vaccine Against the Regulatory Peptide Adrenomedullin Reduces Angiogenesis and Tumor Burden in a Subcutaneous Melanoma Model Without Inducing an Immunosuppressive Tumor Microenvironment

**DOI:** 10.3390/ijms262110745

**Published:** 2025-11-05

**Authors:** Srdan Tadic, Josune García-Sanmartín, Judit Narro-Íñiguez, Alfredo Martínez

**Affiliations:** Angiogenesis Unit, Oncology Area, Center for Biomedical Research of La Rioja (CIBIR), 26006 Logroño, Spain; srdant@riojasalud.es (S.T.); jgarcias@riojasalud.es (J.G.-S.); jnarro@riojasalud.es (J.N.-Í.)

**Keywords:** adrenomedullin, angiogenesis, mRNA vaccine, melanoma, subcutaneous tumor, lipid nanoparticles, cancer, cancer immunotherapy

## Abstract

Adrenomedullin (AM) is a regulatory peptide that stimulates proliferation, migration, and invasion of melanoma cells, and promotes neovascularization within the tumor microenvironment, making it a compelling therapeutic target in melanoma and other cancers. As a continuation of our previous study on a metastatic tumor model, here we tested an mRNA vaccine encoding a fusion antigen comprising keyhole limpet hemocyanin (KLH) and mouse AM in a subcutaneous melanoma mouse model. In vitro synthesized mRNA was encapsulated in lipid nanoparticles (LNPs) and administered to C57BL/6J mice; empty LNPs served as negative controls. After a four-dose immunization schedule, B16-F10 melanoma cells were injected subcutaneously, followed by a fifth immunization. Mice were sacrificed once tumors reached humane endpoints. Immunization led to a significant increase in anti-AM IgG titers (*p* = 0.033) and CD8^+^ T cell (*p* = 0.049) numbers in treated mice. Tumor initiation was significantly delayed (*p* = 0.005) and subcutaneous tumor volume was reduced (*p* = 0.0004) compared to controls. A marked decrease in the area occupied by tumor blood vessels (*p* = 0.028) was also observed, with no signs of systemic toxicity or weight loss. In addition, there was no significant impairment of Ki67^+^ tumor cell proliferation nor changes in the tumor infiltration of CD4^+^, CD8^+^, FoxP3^+^ nor Arg1^+^ cells. The vaccine also proved highly stable at 4 °C, in the absence of cryoprotectants, for more than a month. In summary, we confirmed that a KLH-AM mRNA vaccine is very stable and can elicit humoral and cellular immune responses, inhibit angiogenesis, and delay tumor growth in subcutaneous melanoma, without inducing an immunosuppressive tumor microenvironment (TME), further supporting mRNA vaccines targeting AM as an attractive immunotherapeutic approach.

## 1. Introduction

Despite substantial progress in diagnostics and therapeutic interventions over recent decades, cancer continues to represent one of the most pressing global health challenges. This disease’s seriousness is reflected in the data from 2022, where 10 million deaths were reported as a consequence of cancer, with more than 90% of cancer-related deaths attributed to the consequences of metastasis [1,2].

Nevertheless, while the spread of cancer to vital organs and systemic consequences of metastatic disease lead to death in the majority of cancer patients, the early stages of cancer development, and, in particular, in situ tumors confined to the tissue of origin, represent a critical therapeutic window for intervention [3]. At this stage, malignant cells are localized, surgical excision remains curative in many cases, and therapeutic approaches could successfully avert disease dissemination. Preventing the transition from localized tumors to disseminated disease is therefore an essential strategy to reduce overall cancer mortality [4].

A central process supporting both tumor expansion and metastatic progression is angiogenesis, the sprouting of new capillaries from pre-existing vessels [5]. Tumors rely on angiogenesis to secure sufficient access to oxygen and nutrients while simultaneously overcoming hypoxic microenvironments that arise as the tumor mass increases. Angiogenesis also facilitates metastatic dissemination by providing circulation entry routes and producing an immunosuppressive vascular niche [6]. Under physiological conditions, angiogenesis is tightly regulated by a balance of pro- and anti-angiogenic molecules, maintaining vascular stability in most adult tissues. In tumors, however, this balance is disrupted and pro-angiogenic signals dominate, switching on the “angiogenic switch” [7,8].

Among the most relevant pro-angiogenic factors, such as VEGF and bFGF, induced by tumor hypoxia, we also find adrenomedullin (AM) [9,10]. Inside cells, oxygen sensing is achieved by hypoxia-inducible factor-1 (HIF-1), which is activated under low-oxygen conditions, promoting transcriptional upregulation of pro-angiogenic genes, including VEGF and AM. AM is a 52-amino acid peptide (50 in rodents) belonging to the calcitonin/calcitonin gene-related peptide family, synthesized along with the proadrenomedullin N-terminal 20 peptide (PAMP) as part of a larger precursor, preproadrenomedullin (185 amino acids in humans) [11]. It exerts its pro-angiogenic functions through receptor complexes composed of the calcitonin receptor-like receptor (CLR) and receptor activity-modifying proteins (RAMP2 or RAMP3), generating AM1 and AM2 receptors, respectively [12].

The biological activities of AM extend beyond vascular growth. AM acts as an autocrine/paracrine growth factor, promoting tumor cell motility, stimulating metastasis, preventing apoptosis, and contributing to immune evasion [13]. Elevated AM expression has been reported in numerous solid tumors, including breast, gastrointestinal, liver, and skin cancers [14]. In melanoma, high AM levels correlate with greater vascular density and more aggressive tumor behavior [15]. These findings underscore AM as a critical mediator of tumor progression and an attractive therapeutic target.

Despite extensive efforts in clinical oncology, current anti-angiogenic therapies, such as monoclonal antibodies against VEGF or small-molecule tyrosine kinase inhibitors, offer only limited benefit. Resistance development, adverse side effects, and incomplete efficacy restrict their success [16]. Consequently, a major research focus has shifted toward cancer immunotherapy. Cancer vaccines, in particular, represent an appealing strategy: they elicit adaptive immune responses against tumor-associated antigens, potentially resulting in long-lasting tumor control and prevention of recurrence [17]. Angiogenesis-associated molecules are ideal vaccine targets due to their limited expression in normal adult tissues, broad overexpression across different tumor types, and high genetic stability of endothelial cells [18,19].

Messenger RNA (mRNA) vaccines, meanwhile, have recently emerged as a transformative technology in vaccinology. With high translational efficiency, rapid adaptability, robust immune activation, and favorable safety profiles, mRNA vaccines represent an especially attractive platform for cancer immunotherapy [20]. Despite their widespread application, mRNA cancer vaccines targeting angiogenesis-associated factors, such as AM, remain underexplored.

Melanoma provides a compelling model for studying angiogenesis-targeted immunization’s therapeutic and preventive potential. Melanoma, a malignancy originating from melanocytes, remains a significant contributor to cancer-related mortality due to its aggressive proliferation, ability to evade the immune system, and high metastatic potential [21]. The transition from early, localized melanoma (in situ), where malignant cells are confined to the epidermis, to metastatic melanoma, characterized by dissemination to distant organs, such as lungs and liver, marks a critical shift in tumor biology and clinical prognosis. In situ melanoma often responds well to local treatment, whereas metastatic melanoma presents major therapeutic challenges and poor outcomes [22]. Understanding these biological differences is essential to tailor stage-specific therapeutic approaches. AM has an important role in promoting the proliferation, migration, and invasion of melanoma cells and, furthermore, neovascularization within the tumor microenvironment (TME), making it an appealing target for melanoma therapy [15].

Building on previous work demonstrating that an mRNA vaccine targeting AM effectively reduced tumor burden, angiogenesis, and metastatic spread in a lung metastasis melanoma model [23], this study aimed to extend those findings by evaluating the vaccine’s efficacy in a subcutaneous melanoma model mimicking the in situ tumor setting. This approach allows examination of vaccine effects in early-stage melanoma, where the tumor microenvironment and immune dynamics differ from metastatic disease [24], when intervention could act preventively to suppress future progression. Moreover, translational challenges such as vaccine stability under non-cryopreserved storage conditions are being addressed, supporting feasibility for clinical application.

## 2. Results

Briefly, the KLH-AM mRNA, comprising modified nucleotides and encapsulated into LNPs (vaccine), was administered to one group of animals (treated), via intramuscular injection in the rear hind muscles, while control animals received empty LNPs. Four immunizations, two weeks apart, were given to animals, followed by tumor challenge by subcutaneous injection of B16-F10 melanoma tumor cells. Three days after the tumor challenge, the animals were immunized one more time, and tumor development was followed by volume measurements. Once the tumors reached a humane endpoint, mice were sacrificed, and the blood, tumors and organs of interest were isolated and further analyzed.

### 2.1. The Vaccine Is Highly Stable Even in the Absence of Cryoprotectants

**Figure 1:**
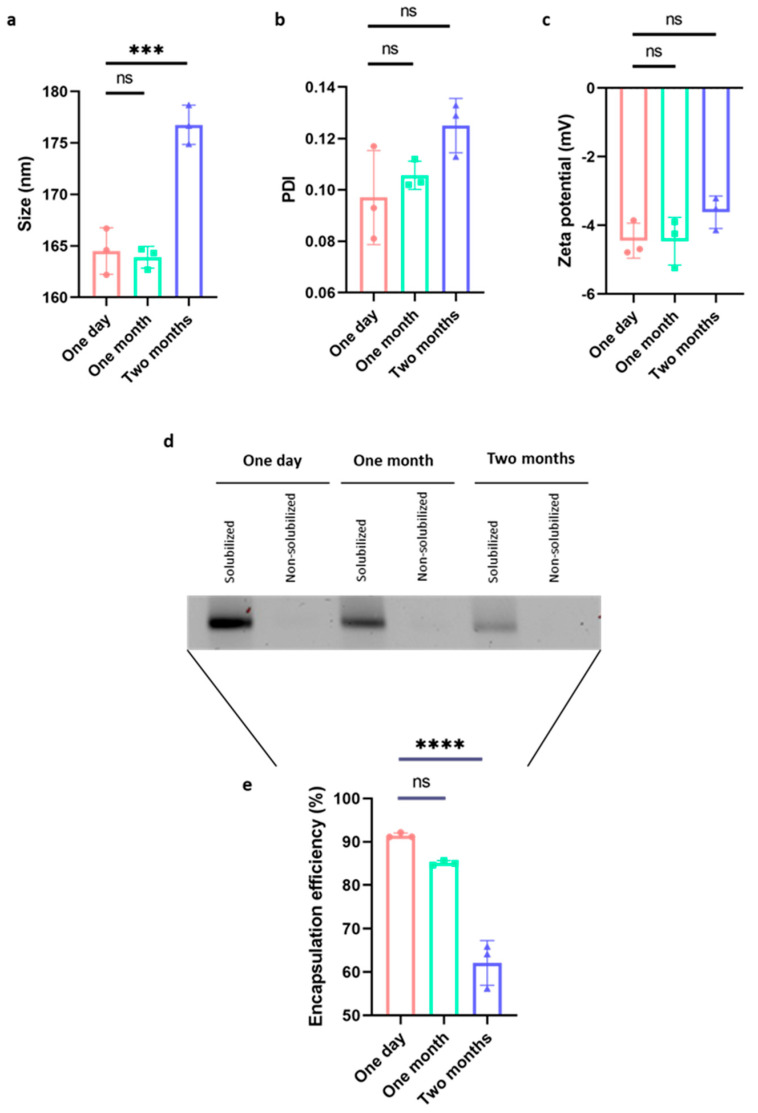
Analysis of the vaccine’s physicochemical characteristics following storage. A significant change in size (**a**), but not in PDI (**b**) or zeta potential (**c**), was demonstrated after two months of storage at 4 °C. Representative image of a 1.0% agarose gel run with KLH-AM mRNA vaccine samples stored at 4 °C without cryoprotectants for different times, and then either solubilized or non-solubilized (**d**), and quantification of the KLH-AM mRNA vaccine encapsulation efficiency indicating a significant reduction after two months of storage at 4 °C without cryoprotectants (**e**). Data analyzed with One-way ANOVA, followed by multiple comparisons represented as Mean ± SD (*** *p* < 0.001; **** *p* < 0.0001; ns = non-significant).

One of the crucial factors influencing vaccine translatability, besides price and ease of use, is its stability. Therefore, we investigated the stability of our mRNA vaccine construct without the addition of any cryoprotectant. Triplicate samples of the vaccine used for the immunization (LNPs encapsulating KLH-AM mRNA) were kept at 4 °C for one day, one month, and two months. After these periods, samples were tested for several physicochemical parameters including size, polydispersity (PDI) and zeta potential, as well as encapsulation efficiency. We observed that the size of the LNPs changed slightly after two months in storage, showing some significant swelling (*p* < 0.001, Figure 1a). On the other hand, there were no significant changes in PDI and zeta potential (Figure 1b,c). In terms of encapsulation efficiency, the vaccine remained highly encapsulated for one month. However, after two months, a significant reduction in encapsulation efficiency was observed (*p* < 0.0001, Figure 1a,b).

### 2.2. Mouse Immunization Induced a Humoral and Cellular Immune Response in the Absence of Measurable Toxicity

**Figure 2:**
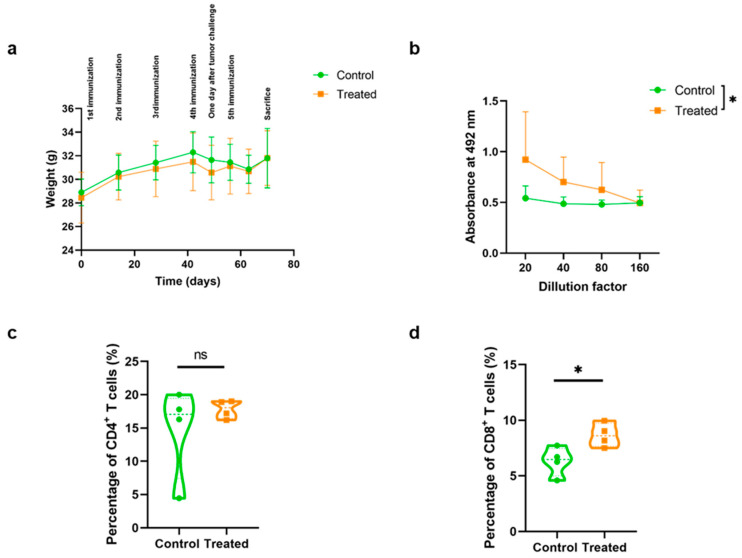
Humoral and cellular immune responses were induced by the vaccine. Animal weight over time (**a**); anti-AM antibody titers by ELISA (**b**); percentage of CD4-positive T cells (**c**); and percentage of CD8-positive T cells (**d**) of both experimental groups. Multiple linear regression (**a**,**b**), and Mann–Whitney-U (**c**,**d**) tests. Data are represented as Mean ± SD (**a**,**b**) or as violin plots (**c**,**d**) of all animals (*n* = 4) (* *p* < 0.05; ns = non-significant).

To test the immunogenicity and safety of the vaccine, animals were immunized i.m. five times at two-week intervals. Considering the severity of the tumor model and the potential detrimental effect that it could have on the animals’ health, all animals were periodically followed for signs of any unusual behavior that could indicate health deterioration such as locomotion difficulties or weight loss. No unusual behavior was observed, indicating a good health status for the mouse colony. However, in one animal of the control group, due to the large size of the tumor, a wound was identified on the tumor surface, and this mouse had to be sacrificed ahead of time. During the immunization campaign, animals were weighed frequently, and no significant differences were observed in their weight (Figure 2a), suggesting a lack of major toxicity.

Following sacrifice, blood and spleens were isolated and analyzed with ELISA (sera) and flow cytometry (splenocytes). We observed that the immunization induced a statistically significant increase (*p* = 0.033) in anti-AM IgG antibody titers in treated animals as compared to controls (injected with empty LNPs) (Figure 2b). This was accompanied by a significant increase (*p* = 0.049) in the percentage of CD8^+^ splenocytes in treated animals as compared to controls (Figure 2d). On the other hand, the percentage of CD4^+^ splenocytes did not change (Figure 2c). The gating strategy is provided in Supplementary Figure S1.

### 2.3. Mouse Immunization Resulted in a Delay in Tumor Initiation and Progression

**Figure 3:**
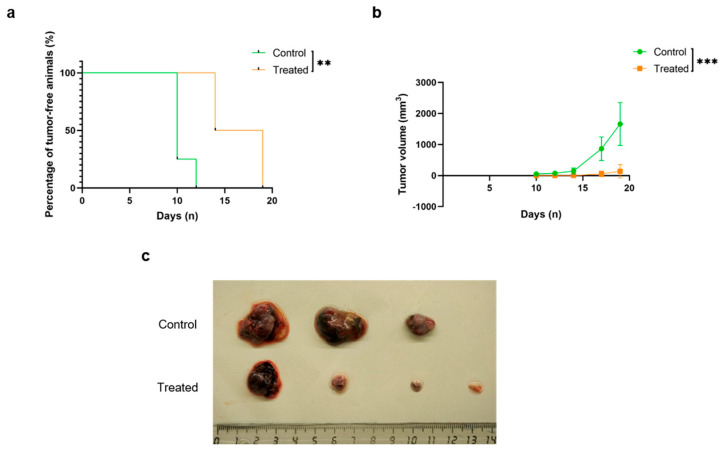
Vaccination resulted in a delay in tumor initiation and progression. Analysis of the appearance of palpable tumor formations (**a**) and tumor volume (**b**) shows significant differences between treated and control animals; photograph of isolated s.c. tumors (**c**) from the control animals (upper) and the treated animals (bottom). The log-rank test (**a**) and Multiple linear regression (**b**) statistical test applied and results represented as Mean ± SD of all animals (*n* = 4) (** *p* < 0.01, *** *p* < 0.001).

After four rounds of immunization, all animals were tumor challenged by injecting 5 × 10^4^ B16-F10 tumor cells subcutaneously in their flanks. The injection sites were observed for signs of palpable tumor development and the date of tumor appearance was recorded, showing a significant delay (*p* = 0.005) in the animals receiving the vaccine (Figure 3a). After reaching a palpable size, tumors were periodically measured with a caliper. As previously commented, one of the control animals got an open wound over its tumor and had to be sacrificed ahead of time. When some tumors reached a volume of 2000 mm^3^, a humane endpoint, all animals were sacrificed, and the tumors were extracted and macroscopically and microscopically analyzed.

The following of tumor growth over time showed that the tumors of the animals immunized with the KLH-AM mRNA vaccine grew significantly more slowly (*p* = 0.0004) than those in the control group (Figure 3b). This was in agreement with the postmortem images of the tumors (Figure 3c) although, due to the loss of one of the control tumors, it was not possible to statistically evaluate tumor weight. These results suggest that the vaccine was able to impair and delay B16-F10 in situ tumor formation, thus confirming a potent anti-tumor effect. To make sure the small number of animals used in this study was enough, we calculated the power of the tumor volume dataset. The mean volume for control animals was 1662.8 mm^3^ and for treated animals 137.9 mm^3^. The analysis resulted in a power of 0.999, or 99.9%.

### 2.4. Mouse Immunization Resulted in a Reduction in Tumor Angiogenesis but Not in Tumor Cell Proliferation

**Figure 4:**
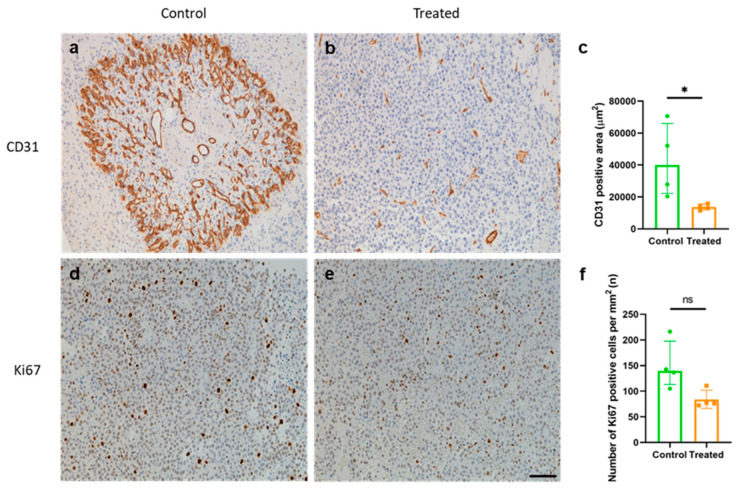
Effects of the vaccine on angiogenesis and tumor cell proliferation. Representative images of tumors isolated from a control (**a**,**d**) and a treated animal (**b**,**e**), stained with CD31 (**a**,**b**) or Ki67 (**d**,**e**) antibody. Quantification of CD31-positive blood vessel area (**c**) shows a significant difference between treated and control animals, while no difference was observed in the number of Ki67-positive cells (**f**). Mann–Whitney-U test applied, bars represent Median ± IQR of all samples (* *p* < 0.05; ns = non-significant). Scale bar = 75 µm.

The anti-angiogenic and anti-proliferative effects of the vaccine were assessed by staining tumor sections against CD31 and Ki67. The immunization with the KLH-AM mRNA vaccine was able to induce a significant decrease (*p* = 0.028) of the CD31^+^ blood vessel area as compared to controls (Figure 4a–c), while no statistically significant difference was observed in terms of Ki67^+^ cells between the experimental groups (Figure 4d–f).

### 2.5. Correlation Studies Indicate a Significant Correlation Between Antibody Titers and a Decrease in Tumor Volume and Blood Vessel Density, but Not with Tumor Proliferation

**Figure 5:**
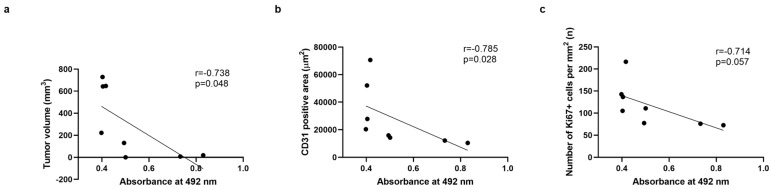
Correlation studies. Correlation study between antibody titer values (absorbance) and tumor volume (**a**), surface of CD31^+^ blood vessels (**b**) or with the number of Ki67^+^ cells per mm^2^ of tumor tissue (**c**). Application of a non-parametric Spearman correlation coefficient analysis (Two-tailed *p*-value analysis (*p* < 0.05; *n* = 8)).

Considering the obtained results, we decided to test whether there was a correlation between antibody titer values and tumor volume, area occupied by blood vessels (CD31^+^ cells) and tumor cell proliferation index (Ki67^+^ cells) in both treated and control animals (Figure 5). We could observe a significant inverse correlation between antibody titer values and the tumor volume (r = −0.738; *p* = 0.048; Figure 5a) as well as the area occupied by CD31^+^ cells (r = −0.785; *p* = 0.028; Figure 5b) inside tumor tissues of animals involved in the study, while the same was not observed for the number of Ki67^+^ cells (Figure 5c).

### 2.6. Immunization with KLH-AM Did Not Induce Changes in the Number of Tumor-Infiltrating CD4^+^ or CD8^+^ T Cells

**Figure 6:**
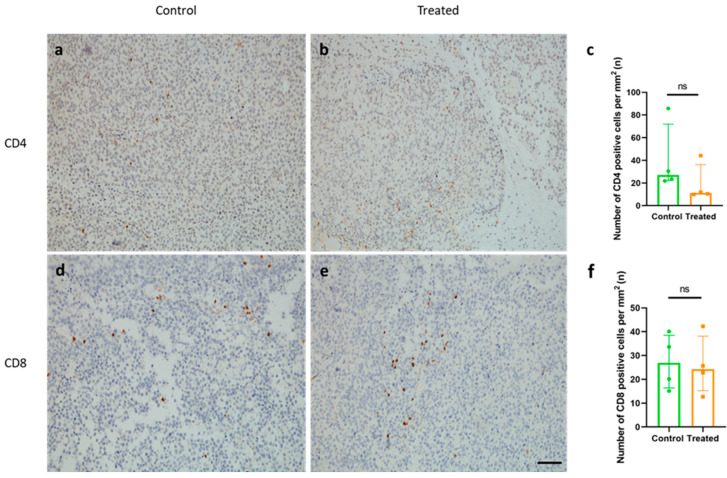
Vaccination did not influence CD4^+^ or CD8^+^ T cell tumor infiltration. Representative images of tumor sections from a control (**a**,**d**) and treated animal (**b**,**e**), stained with CD4 (**a**,**b**) and CD8 (**d**,**e**) antibodies. Quantification of CD4^+^ cells (**c**) and CD8^+^ cells (**f**) shows no significant differences between groups. Mann–Whitney-U test applied, bars represent Median ± IQR of all samples (ns = non-significant). Scale bar = 75 µm.

In melanoma, an important prognostic factor is the presence of tumor infiltrating lymphocytes (TILs) [25]. Thus, to test whether the immunization with KLH-AM mRNA vaccine was able to modulate lymphocyte infiltration, we stained tumor sections with antibodies against CD4 and CD8. No significant differences in CD4^+^ nor in CD8^+^ T cell numbers were observed inside tumor tissue between both experimental groups (Figure 6).

### 2.7. Immunization with KLH-AM Did Not Change the Features of the Immunosuppressive TME

**Figure 7:**
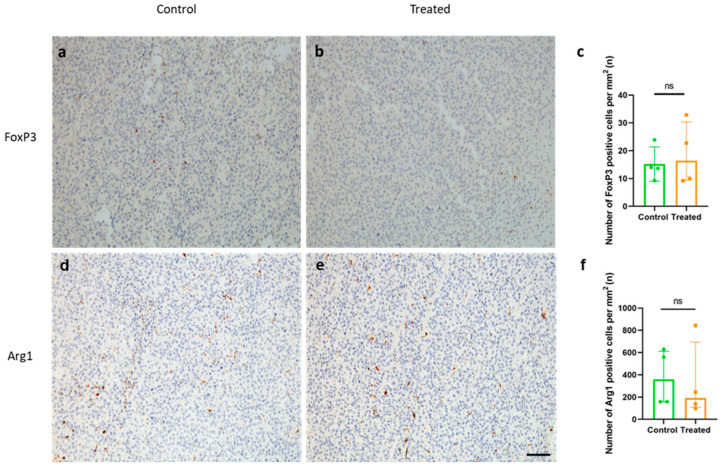
Vaccination did not modify the number of FoxP3- or Arg1-positive cells inside the tumors. Representative images of tumors isolated from a control (**a**,**d**) and a treated animal (**b**,**e**), stained with FoxP3 (**a**,**b**) and Arg1 (**d**,**e**) antibodies. Quantification of FoxP3^+^ cells (**c**) and Arg1^+^ cells (**f**) shows no difference between treated and control animals. Mann–Whitney-U test applied, bars represent Median ± IQR of all samples (ns = non-significant). Scale bar = 75 µm.

Another important feature of melanoma is the establishment of an immunosuppressive TME and the presence of predominantly immunosuppressive cell subtypes, such as tumor-associated macrophages (TAMs) [26] and regulatory T cells (Tregs) [27], which are generally related to poorer clinical outcomes. To analyze the effect of the vaccine on the modulation of the immunosuppressive TME, tumor sections were analyzed for the presence of infiltrating Tregs, using FoxP3 as a marker (Figure 7a,b), and of TAMs, using Arg1 as a marker of M2 macrophages (Figure 7d,e). No significant differences in the number of either FoxP3 or Arg1 positive cells inside tumors were found between groups (Figure 7c,f).

## 3. Discussion

This study demonstrates that a KLH-AM mRNA vaccine elicits strong humoral and cellular immune responses and effectively inhibits melanoma tumor angiogenesis and growth in a subcutaneous mouse model. The significant increase in anti-AM IgG titer and CD8^+^ T cell activation confirms the vaccine’s ability to induce a specific immune response targeting AM, one of the critical regulators of tumor angiogenesis and tumor progression. The delay in tumor initiation and progression observed in vaccinated mice align with our therapeutic hypothesis that targeting AM disrupts crucial pathways promoting tumor growth. Importantly, the absence of systemic toxicity or weight loss underscores the vaccine’s favorable safety profile, supporting its further development as a cancer immunotherapeutic approach.

As stated before, AM represents an important pro-angiogenic factor that, through interaction with its receptors on the endothelial cells, initiates angiogenesis, which furthermore promotes the transport of nutrients and other factors, thus providing energy for tumor growth and metastasis [14]. In this study, we demonstrated that targeting AM with our mRNA vaccine formulation was able to significantly decrease the area occupied by CD31^+^ blood vessels, thus indicating significant impairment of tumor-related angiogenesis. However, although known as an autocrine tumor growth factor [28], targeting of AM in our study did not induce significant differences in the tumor cell proliferation index, as analyzed by the number of Ki67^+^ tumor cells per mm^2^, thus suggesting that the main effect induced by our vaccine in this tumor model was anti-angiogenic.

Furthermore, the results of the correlation studies indicated that the vaccine effect on tumor volume and the area occupied by blood vessels inside tumors significantly correlates with the level of antibody titers induced in the vaccinated. This can be attributed to AM being secreted into the extracellular environment, where it can perform its activity via receptors present on the surface of the cells (either tumoral of endothelial) or via soluble molecules such as complement factor H [13,29]. Thus, the induced humoral immune response could potentially block AM binding to these receptors thus leading to the impairment of its tumor-related pro-angiogenic functions. Similar effects have been shown by different studies where antibodies against AM were injected into the animals [30], including reduction in intratumoral angiogenesis and tumor volume, thus confirming the therapeutic effect of anti-AM antibodies either generated by the vaccine or administered as an external drug.

Considering the significant increase in CD8^+^ T splenocytes in treated (KLH-AM vaccine) animals, we decided to further investigate cellular immune response features inside the tumors. Additionally, it is known that AM fosters a tolerogenic TME by promoting the expansion of Treg cells, impairing the function of dendritic cells, and promoting the pro-tumorigenic function of mast cells in other tumor models [31,32,33], so we were expecting a modulatory effect of our vaccine on the TME. Although immunization with the KLH-AM mRNA vaccine induced a significant increase in CD8^+^ T cell spleen cell population as compared to control, our results indicate a lack of significant changes in tumoral infiltration by immune cells such as CD8^+^, CD4^+^, Tregs, or tumor-associated macrophages (Arg1^+^). This furthermore suggests that, although the vaccine successfully induces CD8^+^ T cell activation and modulates angiogenesis by targeting AM, it does not sufficiently alter the broader immunosuppressive TME. The lack of effect on the immune cells’ composition within the tumor can be possibly attributed to several factors, such as the established immunosuppressive environment, compensatory mechanisms within the tumor, and the prominent features of the tumor model, which is considered extremely aggressive [34,35,36]. However, besides quantity, the functionality of these cells is crucial for their effects inside the TME; for instance, the exhaustion profile of the CD8^+^ T cells with high PD-L1 expression contributes to poorer outcomes in cancer patients [37]. Future studies should therefore explore the functional characteristics of these cell types in greater detail, as well as increase the marker pool, to draw more clinically relevant conclusions regarding the vaccination’s effect on TME immunosuppression.

Interestingly, a recently published study by our group indicated that a DNA vaccine targeting another peptide generated from the same precursor molecule as AM, PAMP, although not able to reduce tumor growth, induced high infiltration of different immune cells, including Tregs and M2 macrophages, in mouse tumors [38], thus suggesting quite distinct mechanisms of action related to the immune response of these two apparently related targets.

Due to the rapid growth of the syngeneic B16-F10 tumor model [39], this study used a prophylactic vaccination approach, immunizing mice before tumor challenge. Considering the vaccine was not yet tested in a therapeutic settings and considering results of the study, we propose the potential application of this vaccine as an additional, anti-angiogenic therapy, for patients with active cancer or as a preventive measure for those at high risk of recurrence after primary tumor treatment [40]. However, although effective when applied by itself, in a clinical setting, the vaccine is more likely to be combined with other cancer treatment modalities. For instance, given the lack of a modulatory capacity of the TME, our vaccine would benefit from a combination with drugs that can induce significant infiltration of potent anti-tumoral immune cells, such as killer T cells inside the tumor, and thus improve its general and long-term effectiveness. These treatments include chemotherapy, radiotherapy, or immunotherapy, aligning with current antiangiogenic treatment strategies [41].

For example, an mRNA cancer vaccine named “autogene cevumeran” encoding up to 20 neoantigens, developed by BioNTech, is used in combination with atelozolizumab (an anti-PDL1 antibody), and a chemotherapy regimen comprising four drugs (folinic acid, fluorouracil, irinotecan, and oxaliplatin, also called mFOLFIRINOX), and was assessed in a phase 1 clinical trial on patients with resected pancreatic ductal adenocarcinoma (PDAC). This vaccine induced high levels of long-lived neoantigen-specialized T cells targeting multiple neoantigens. In addition, the responders had longer recurrence-free survival, suggesting that this vaccine induced an efficient anti-tumor immune response [42]. More recent publications regarding the same trial indicate that patients developed a strong memory T cell response, preventing tumor recurrence [43].

Although in both of our studies no visible toxicity was observed, it should be noted that translating anti-angiogenic immunotherapy findings into clinical use requires balancing efficacy and safety, especially when targeting molecules such as AM with roles beyond the TME. A major concern is off-target effects on normal vessels, causing issues such as impaired wound healing or hypertension, as seen with anti-VEGF therapies [44,45]. Surprisingly, most studies of DNA vaccines targeting angiogenic factors report no serious vascular toxicity, suggesting complex mechanisms and a high safety profile [46]. Also, our previous report showed no deleterious effects of the vaccine on normal blood vessels [23] underlying the vascular safety of the treatment. Several features of AM and of the tumor vasculature potentially contribute to these unexpected but encouraging results. For instance, *Adm* knockout studies show that eliminating AM before the critical stages of embryo angiogenesis results in 100% embryo lethality [47], but in adult mice, inducing a full-body AM knockout produces only mild effects, indicating that anti-AM treatments in adults may show limited adverse effects [48]. AM is a promising alternative target to VEGF since it can induce VEGF expression and is upregulated in tumors resistant to anti-VEGF treatments [49,50,51]. The tumor vasculature’s distinct, fragile nature makes it more susceptible to anti-angiogenic therapy, sparing normal vessels [19,52]. Nevertheless, AM’s roles in cardiovascular regulation, reproduction, placental development, and neuroendocrine signaling add complexity and additional potential toxicity targets [53,54]. Therefore, the assays such as wound healing, biochemistry/hematology (toxicological blood parameters) and/or blood pressure, as well as dosage should be further investigated and carefully optimized in future studies aiming to improve potential for clinical translatability. Furthermore, prophylactic vaccine use should exclude pregnant patients and those with bleeding or wound-healing issues.

An important feature of mRNA vaccines is their stability. Initial formulations of mRNA vaccines, such as those targeting COVID-19, had difficulties overcoming degradation and short storage life. However, newer formulations, due to more stable lipid formulations and use of cryoprotectants, can last longer, but, for the most part, they still require low storage temperatures, including −80 °C in some cases [55,56]. The demonstrated stability of our mRNA vaccine formulation at 4 °C without cryoprotectants for over a month provides a practical advantage for storage and distribution and a reduction in cost, aligning with current advances in mRNA vaccine technologies emphasizing cold-chain management and scalability [57]. However, a more detailed analysis of the vaccine should be done in the future to assess its functionality, such as immunogenicity and anti-tumor function, under these conditions. Additionally, the vaccine could possibly be improved by using novel lipid formulations and/or lyophilization methods [58], thus enhancing its potential.

Despite the promising results, this study has several limitations. Since this was a proof-of-concept study, the experiments were conducted solely in male C57BL/6J mice, which may introduce sex bias and limit the generalizability of findings given known differences in tumor growth and immune response characteristics between male and female mice [59]. Future studies need to include both sexes. Additionally, the use of a single tumor model limits the broader translatability of results. For instance, it should be noted that the B16-10 melanoma model, although generally used for initial testing of cancer therapeutics, also suffers from extremely rapid advancement, thus making it hard to treat with vaccine modalities [36,60]. Additionally, the lack of immune-mediated cytotoxic effect of CD8^+^ T cells present in tumors of treated animals, as indicated by the lack of a reduction in the number of Ki67^+^ cells, could possibly be attributed to the B16-F10 tumor model features, considering that these cells suffer from low levels of major histocompatibility complex I (MHCI) markers, thus impacting the presentation of tumor antigens and consequently initiation of the cellular anti-tumor response [61]. Furthermore, significant differences in survival or immune profile of tumors between males and females can be tumor type-dependent, thus adding an additional layer of complexity to the choice of the most appropriate tumor model [62]. Thus, the use of other more advanced models in the future, which allow “wider” treatment windows, could provide a deeper understanding of the vaccine effects. Furthermore, although the results of this study and a previous one demonstrate high specificity of the immune response against AM per se as a consequence of vaccination, we cannot exclude the contribution of, for instance, a KLH-driven response. Considering the KLH potency as an immunogenic carrier, in future studies the addition of a control LNP vehicle comprising only KLH sequence should be done aiming to provide further insight. Furthermore, although the timeline of tumor initiation was analyzed, it would be very important to analyze the survival of the animals and perform long-term assessments such as memory immune responses or tumor recurrence, which are crucial in the cancer therapy context [63].

## 4. Materials and Methods

### 4.1. Transfection of E. coli, Amplification and Linearization of the DNA Template

The expression vector was previously described [23] and was inserted into competent *E. coli* following standard heat shock protocols and selection in ampicillin plates. The vector was purified using the Plasmid DNA MaxiPrep kit (Qiagen, Hilden, Germany) following manufacturer’s instructions. The DNA template was linearized using BssHII restriction enzyme (NewEngland BioLabs, Ipswich, UK) and purified by isopropanol precipitation. The linearized template was resuspended in HyClone HighPure water (Cytiva, Marlborough, MA, USA).

### 4.2. In Vitro Transcription, Capping and Characterization

The therapeutic mRNA was synthesized by in vitro transcription (IVT) using T7-Scribe™ Standard RNA IVT Kit (CellScript, Madison, WI, USA) to produce standard/non-modified mRNA. Alternatively, for in vivo experiments, modified mRNA was generated by using the INCOGNITO™ T7 5mC- & Ψ-RNA Transcription Kit (CellScript), following manufacturer’s instructions. Briefly, the linearized DNA template was mixed with components of the kit, and the reaction was incubated at 37 °C for 2 h. Then, DNase I was added to remove traces of the DNA template. The reaction product was purified by standard ammonium acetate precipitation and dissolved in RNAse-free water. The amount and purity of the RNA were analyzed on a NanoDrop spectrophotometer (Thermo Fisher Scientific, Waltham, MA, USA).

Capping of modified IVT-mRNA was done post-transcriptionally using the ScriptCap™ Cap 1 Capping System (CellScript). The Cap1 structure was added by using both ScriptCap Capping Enzyme, and ScriptCap 2′-O-Methyltransferase, according to manufacturer’s instructions. The reaction was performed at 37 °C for 2 h. The quality of the obtained capped mRNA was assessed with Nanodrop and Qubit (Thermo Fisher Scientific).

### 4.3. In Vitro Transcription Encapsulation of the mRNA in Lipid Nanoparticles

LNPs were prepared using the ionizable lipid, 4-(dimethylamino)-butanoic acid, (10Z,13Z)-1-(9Z,12Z)-9,12-octadecadien-1-yl-10,13 nonadecadien-1-yl ester (DLin-MC3-DMA, Cayman Chemical, Ann Arbor, MI, USA). In addition, three helper lipids were added, including disteraroylphosphatidylcholine (DSPC, Avanti Polar Lipids, Alabaster, AL, USA), 1,2-dimyristoyl-rac-glycero-3-methoxypolyethylene glycol (DMG-PEG 2000, Cayman Chemical), and cholesterol (Thermo Fisher Scientific). The molar ratio of lipids, DLin-MC3/Chol/DSPC/PEG, was 50/10/38.5/1.5. To generate the particles, lipids were dissolved in ethanol whereas the mRNA was dissolved in citrate buffer (pH 4.0). These solutions were mixed at a 1:3 volume ratio, to achieve a 40:1 weight lipid/mRNA ratio, by rapid pipetting [64]. Constructs were allowed to stabilize for 15 min and then were transferred to Amicon Ultrafilter tubes, where they were centrifuged at 13,000× *g*, at 4 °C, for 30 min. The flow-through was kept for subsequent analyses, whereas the solution remaining inside the tubes was transferred to new tubes and centrifuged at 13,000× *g*, at 4 °C, for 2 min. Nanoparticles were in the pellet of the last spin. Control LNPs were prepared following the same protocol in the absence of mRNA.

### 4.4. Characterization of Lipid Nanoparticles Following Long-Term Storage

LNPs encapsulating KLH-AM mRNA and empty (control) LNPs were stored without cryoprotectants at 4 °C for one day, one month, and two months. After these periods, samples were tested for physicochemical parameters such as size, PDI and zeta potential, as well as for encapsulation efficiency.

Size, polydispersity (PDI), and zeta potential of LNPs encapsulating or not (control) mRNA were analyzed with Zetasizer (Malvern, Worcestershire, UK) according to manufacturer’s instructions. Briefly, samples were diluted in PBS and injected into specialized capillaries. Samples were transferred to standard cuvettes, and their size, PDI and Zeta potential were measured. Six measurements were taken per sample, and the mean and standard deviation were calculated.

To determine encapsulation efficiency, LNPs (both empty and containing mRNA) were separated in two groups; LNPs in Group 1 were solubilized with 2% Triton X-100 and Tris-EDTA and heated at 37 °C for 10 min while shaking. On the other hand, LNPs in Group 2 were not solubilized. Naked mRNA was used as a control. Finally, all samples were electrophoresed in 1.0% agarose gels and images recorded with a gel imaging system (BioRad, Hercules, CA, USA).

Furthermore, fluorimetric measurements were performed by using a high-sensitivity kit for the detection of RNA (HS RNA, Invitrogen, Carlsbad, CA, USA) and a Qubit fluorometer (Thermo Fisher Scientific) following manufacturer’s instructions. Final calculations were done based on the amount of mRNA present in solubilized samples divided by the initial mRNA used for encapsulation and multiplied by 100, with the following formula:

mRNAfinal = (mRNAsample/mRNAinitial) × 100

### 4.5. Cell Lines and Culture Conditions

Murine melanoma cell line B16-F10 (RRID CVCL_0159) was obtained from the American Type Culture Collection (ATCC, Manassas, VA, USA) and cultured in Roswell Park Memorial Institute (RPMI 1640, Corning, Manassas, VA, USA) supplemented with 10% fetal bovine serum (FBS, Thermo Fisher Scientific), at 37 °C and 5% CO_2_ in a humidified incubator. The cell line was authenticated by STR profiling (IDEXX BioAnalytics, Kornwestheim, Germany).

### 4.6. Animals and Immunization Protocol

All experiments and animal procedures were carried out following the guidelines laid down by the European Parliament and Counsel (2010/63/UE) and Spanish Royal Decree 53/2013 on animal experimentation, and were revised and approved by the Committee for Animal Welfare of the Center for Biomedical Research of La Rioja (OEBA/CIBIR), Spain, ref. AMR-17.

Group size number calculation was performed by the online sample size calculator (ClinicCal; https://clincalc.com/stats/samplesize.aspx, accessed on 4 May 2025) using the following formula:

$$n1=\frac{\left(\sigma 21+\frac{\sigma 22}{K}\right)\left(z1-\frac{\alpha }{2}+z1-\beta \right)2}{\Delta 2}$$

n2=k ∗ n1

(Δ = |μ2 − μ1| = absolute difference between two means; *σ*1, *σ*2 = variance of mean #1 and #2; *n*1 = sample size for group #1; *n*2 = sample size for group #2; *α* = probability of type I error (usually 0.05); *β* = probability of type II error (usually 0.2); *z* = critical Z value for a given *α* or *β*, *k* = ratio of sample size for group #2 to group #1)

The analysis was based on the data acquired in the literature [65], in which the mean tumor volume difference between control and treated animals was more than 3-fold, with control animals having s.c. tumors of about 1450 mm^3^, and a treated animals of about 450 mm^3^, type I/II error parameters (*α* = 0.05; *β* = 0.2) resulting in a sample size of four animals per group (*n* = 4), and a total of eight animals.

Immunocompetent mice (7-week-old male C57BL/6 mice) were purchased from Envigo (Indianapolis, IN, USA). Animals were housed under specific pathogen-free conditions at the CIBIR animal facility (ES260890000992), in a temperature-controlled room with 12-h light/dark cycle and reared on standard chow and water provided ad libitum. All animals were checked by a veterinarian, and weighed, on a weekly basis, to ensure animal wellbeing and lack of vaccine toxicity.

For the immunization campaign, male C57BL/6 mice were randomly divided into two experimental groups: control (*n* = 4) and treated (*n* = 4). The control group received empty LNPs, while the treated group received LNPs containing the KLH-AM mRNA. Animals were injected intramuscularly (i.m.) in the rear hind muscle with 50 µL of the vaccine containing a total of 5 µg of mRNA (or with empty LNPs for the control). Five immunizations were implemented at two-week intervals, with the last injection given 3 days after subcutaneous (s.c.) tumor challenge with B16-F10 cells. Animals were sacrificed when tumor volume reached a humane endpoint and blood and tissues were collected for further analysis.

### 4.7. Tumor Challenge

To test the antitumor effects of the vaccine, immunized animals (*n* = 8) were challenged with syngeneic B16-F10 melanoma tumor cells. Mice were injected with 5 × 10^4^ B16-F10 cells/animal in the right flank at random to induce tumors. Tumor volume was measured thrice a week using a digital caliper, as previously published [66]. When tumors reached a critical humane endpoint, mice were sacrificed and blood and tissues were collected.

To ensure that the statistical power of the study was sufficient, a post hoc statistical power analysis was performed using a calculator available online (ClinicCal; https://clincalc.com/stats/power.aspx, accessed on 20 August 2025), and the following formula was applied:

$$Power=\Phi \{-Z1-\alpha /2+\frac{\Delta }{\surd \sigma_{2}1/n1+\sigma_{2}2/n2}\}$$

where (*n*1 = sample size for group #1; *n*2 = sample size for group #2; Δ = |μ2 − μ1| = absolute difference between two means; *σ*1, *σ*2 = variance of mean #1 and #2; α = probability of type I error; Z = critical Z value for a given α; Φ = function converting a critical Z value to power).

### 4.8. Serum Characterization (ELISA)

Sera was isolated from blood following standard protocols. For ELISA, COSTAR high-binding 96-well plates (Corning, New York, NY, USA) were coated with 100 ng/50 µL/well of mouse AM peptide (Phoenix Pharmaceuticals, Burlingam, CA, USA), dissolved in carbonate coating buffer pH 9.6, and incubated overnight at 4 °C. The next day, plates were washed with PBS and then blocked with 1.5% FBS in PBS for 1h at RT with shaking. Sera samples (*n* = 8) were serially diluted in PBS (from 1:20 to 1:180), added to wells, as technical triplicates, and incubated at RT with shaking for 2 h. Wells were washed again and incubated with anti-mouse secondary antibody bound to HRP (Table 1) at a 1:10,000 dilution for 1h. After washing again, 3,3′,5,5′-Tetramethylbenzidine (TMB) substrate was added to each well. Color development was stopped with 3N HCl and plates were analyzed on a microplate reader (POLARstar Omega, BMG Labtech, Ortenberg, Germany) at 492 nm. For statistical analysis, per-mouse values were used.

### 4.9. Isolation of Splenocytes

Spleens were collected from immunized animals (*n* = 8), minced into small pieces, and passed through 70 µm cell strainers (Corning). Resulting cells were resuspended in RPMI 1640 media supplemented with 1.0% penicillin/streptomycin (Gibco, Waltham, MA, USA). After three washes to remove cell debris, cells were exposed to Ammonium–Chloride–Potassium (ACK) red blood cell lysis buffer (Thermo Fisher Scientific) on ice. Then several washes were performed until a clear pellet was obtained. Finally, cells were resuspended in RPMI 1640 media supplemented with 10% FBS and 1.0% penicillin/streptomycin.

### 4.10. Flow Cytometry

For cell labeling, aliquots of 1.0 × 10^6^ cells per animal (*n* = 8) were prepared. Staining was performed for surface markers CD45, CD4 and CD8 according to standard protocols. Briefly, cells were resuspended in FACS buffer (2.0% FBS plus 0.01% ammonium azide in PBS). Then, cells were incubated with Fc block (anti-CD16/32, BD Biosciences, Franklin Lakes, NJ, USA) in FACS buffer for 5 min on ice. Cells were then washed, centrifuged at 350× *g* at 4 °C, and the supernatant discarded. Cells were then exposed to an antibody mixture of CD45 PerCP-Cy5.5, CD4 APC-Cy7 and CD8b.2 FITC (Table 1) in FACS buffer and incubated for 30 min on ice, in the dark. Flow cytometry was performed on a FACSCantoII cytometer (BD Biosciences). For statistical analysis, per-mouse values were used. The gating strategy is presented in Supplementary Figure S1.

### 4.11. Morphological and Microscopical Assessment of Resulting Tumors

After dissection, small pieces of the tumors (*n* = 8) were transferred to 10% buffered formalin, fixed for 24 h, and paraffin-embedded. Tissue sections (3 µm-thick) were stained with hematoxylin and eosin and assessed under a light microscope (DM6000B, Leica, Wetzlar, Germany). A double blinding strategy was applied in order to prevent potential bias; stained slides were identified with just a code and researchers ranking morphological variables had no access to treatment information.

### 4.12. Immunohistochemistry

Additional tumor sections, two per animal (*n* = 8) and antibody tested, were rehydrated and antigen retrieval was performed by heating in citrate buffer (pH 6.0) (for CD31 and Ki67) or Tris-EDTA buffer (pH 9.0) (for CD4, CD8, FoxP3 and Arg1) for 20 min at 96 °C. After blocking with normal donkey serum, sections were incubated overnight with primary antibodies including anti-CD31, anti-Ki67, anti-CD4, anti-CD8, anti-FoxP3, and anti-Arg1 (Table 1). The lack of primary antibody was used as a negative control in a serial section. The next day, following several washes in PBS, sections were incubated with a polymer complex containing HRP or secondary IgG HRP conjugated antibody (Table 1). Immunoreactivity was developed with diaminobenzidine (DAB, Dako, Santa Clara, CA, USA). Slides were lightly counterstained with hematoxylin. Pictures were taken in random fields (*n* = 10 per mouse) and the area occupied by the blood vessels per µm^2^ (for CD31) or the number of positive cells per mm^2^ (Ki67, CD4, CD8, FoxP3, and Arg1) was quantified through free digital pathology QuPath software, v. 0.5.1 [67]. For each mouse, the mean of the 10 fields was used as the individual value and statistical analysis was performed based on these values.

### 4.13. Statistics

Considering small number of samples (*n* = 4) all datasets were treated as non-normal. Data were compared with the Mann–Whitney’s U test, Multiple linear regression test, or by Spearman correlation coefficients test. Furthermore, in the experiment where the tumor initiation timing was analyzed, the log-rank test, including 95% confidence intervals, was applied. All these studies were performed with GraphPad Prism version 5.02 (GraphPad Software, Inc., La Jolla, CA, USA). A *p* < 0.05 was considered statistically significant.

## 5. Conclusions

In conclusion, the KLH-AM mRNA vaccine represents a promising prophylactic or additional immunotherapeutic approach for the treatment of primary tumors, such as melanoma, effectively inhibiting tumor angiogenesis and growth with a strong immune activation profile and favorable safety. These preliminary findings support further investigation into the combination of AM with other tumor-associated antigens (TAAs) and tumor-specific antigens (TSAs) in multivalent mRNA vaccine platforms, the combination of AM-targeting mRNA vaccines with complementary therapeutic strategies or standard therapies such as chemotherapy, to maximize clinical benefit in melanoma and potentially other solid tumors. Although the study aimed to assess the utility of AM as a valid target that could be used in mRNA cancer vaccines, future studies incorporating both sexes, larger cohorts, and additional models will be crucial to validate and extend these findings.

## Figures and Tables

**Table 1:** Antibodies used in the study.

Type of Antibody	Antibody	Manufacturer	Cat. No.	RRID	Dilution
Primary	Anti-mouse CD45 PerCP-Cyanine5.5	BioLegend, San Diego, CA, USA	103132	AB_893340	1/100
	Anti-mouse CD4 APC-Cyanine7	BioLegend	100526	AB_312727	1/200
	Anti-mouse CD8b.2 FITC	BioLegend	140404	AB_10643587	1/100
	Anti-mouse CD31	Abcam, Cambridge, UK	ab281583	AB_3096925	1/5000
	Anti-mouse Ki67 (clone SP6)	Vitro Master Diagnostica, New York, NY, USA	MAD-000310QD-3	AB_3677420	1/5
	Anti-mouse CD4	Abcam	ab288724	AB_2941893	1:4000
	Anti-mouse CD8	Abcam	ab217344	AB_2890649	1:2000
	Anti-mouse FoxP3	Thermo Fisher Scientific	14-5773-82	AB_467576	1:100
	Anti-mouse Arg1	Cell Signaling, Danvers, MA, USA	93668	AB_2800207	1:2000
Secondary	Novolink rabbit detection system	Leica, Wetzlar, Germany	RE7200CE	AB_3674357	Prediluted
	Peroxidase-AffiniPure Goat Anti-Rat IgG (H+L)	Jackson Immunoresearch, West Grove, PA, USA	112-035-003	AB_2338128	1:200

## Data Availability

All data are available in the main text or the Supplementary Materials.

## Supplementary Materials

The following supporting information can be downloaded at https://www.mdpi.com/article/10.3390/ijms262110745/s1.

## Abbreviations

The following abbreviations are used in this manuscript:

AM	Adrenomedullin
ANOVA	Analysis of variance
CLR	Calcitonin receptor-like receptor
HIF	Hypoxia inducible factor
IVT	In vitro transcription
KLH	Keyhole limpet hemocyanin
LNP	Lipid nanoparticle
MHCI	Major histocompatibility complex I
PAMP	Proadrenomedullin N-terminal 20 peptide
PDAC	Pancreatic ductal adenocarcinoma
PDI	Polydispersity index
RAMP	Receptor activity-modifying protein
TAA	Tumor-associated antigen
TAM	Tumor-associated macrophage
TIL	Tumor infiltrating lymphocyte
TME	Tumor microenvironment
TSA	Tumor-specific antigen
