# Supplementary material for: A Stable RNA Vaccine Against the Regulatory Peptide Adrenomedullin Reduces Angiogenesis and Tumor Burden in a Subcutaneous Melanoma Model Without Inducing an Immunosuppressive Tumor Microenvironment
Source: Int J Mol Sci. 2025 Nov 5;26(21):10745. doi: 10.3390/ijms262110745 (PMC12609770; doi:10.3390/ijms262110745)
Supplement: Supplementary file 1 [file ijms-26-10745-s001.zip › ijms-3956819-supplementary.pdf]

Supplementary Figure S1

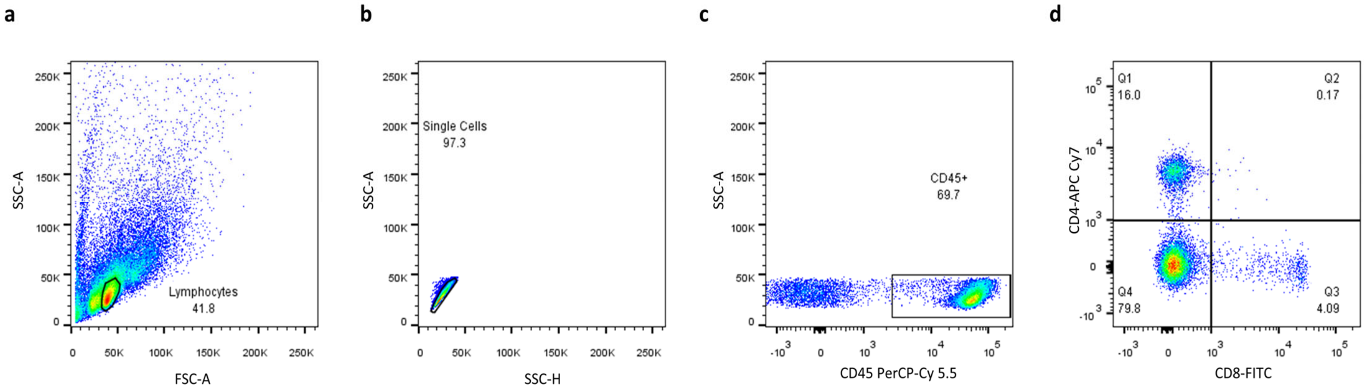

Supplementary Figure S1. Gating strategy applied. Lymphocyte sub-population gated (**a**), single cell sub-population gated (**b**), $CD45^+$ sub-population gated (**c**), $CD8^+$ and $CD4^+$ sub-population gated (**d**). Layout obtained from FlowJo software.
